# Medication errors among nurses in teaching hospitals in the west of Iran: what we need to know about prevalence, types, and barriers to reporting

**DOI:** 10.4178/epih.e2017022

**Published:** 2017-05-17

**Authors:** Afshin Fathi, Mohammad Hajizadeh, Khalil Moradi, Hamed Zandian, Maryam Dezhkameh, Shima Kazemzadeh, Satar Rezaei

**Affiliations:** 1 Department of Pediatrics, Ardabil University of Medical Sciences, Ardabil, Iran; 2 School of Health Administration, Faculty of Health Professions, Dalhousie University, Halifax, Canada; 3 Imam Reza Hospital, Kermanshah University of Medical Sciences, Kermanshah, Iran; 4 Health Management and Economics Research Center, Iran University of Medical Sciences, Tehran, Iran; 5 Student Research Committee, Kermanshah University of Medical Sciences, Kermanshah, Iran; 6 Research Center for Environmental Determinants of Health, Kermanshah University of Medical Sciences, Kermanshah, Iran

**Keywords:** Medication errors, Prevalence, Nurses, Iran

## Abstract

**OBJECTIVES:**

This study aimed to examine the prevalence and types of medication errors (MEs), as well as barriers to reporting MEs, among nurses working in 7 teaching hospitals affiliated with Kermanshah University of Medical Sciences in 2016.

**METHODS:**

A convenience sampling method was used to select the study participants (n=500 nurses). A self-constructed questionnaire was employed to collect information on participants’ socio-demographic characteristics (10 items), their perceptions about the main causes of MEs (31 items), and barriers to reporting MEs to nurse managers (11 items). Data were collected from September 1 to November 30, 2016. Negative binomial regression was used to identify the main predictors of the frequency of MEs among nurses.

**RESULTS:**

The prevalence of MEs was 17.0% (95% confidence interval, 13.7 to 20.3%). The most common types of MEs were administering medications at the wrong time (24.0%), dosage errors (16.8%), and administering medications to the wrong patient (13.8%). A heavy workload and the type of shift work were considered to be the main causes of MEs by nursing staff. Our findings showed that 45.0% of nurses did not report MEs. A heavy workload due to a high number of patients was the most important reason for not reporting MEs (mean score, 3.57±1.03) among nurses. Being male, having a second unrelated job, and fixed shift work significantly increased MEs among nurses (*p*=0.001).

**CONCLUSIONS:**

Our study documented a high prevalence of MEs among nurses in the west of Iran. A heavy workload was considered to be the most important barrier to reporting MEs among nurses. Thus, appropriate strategies (e.g., reducing the nursing staff workload) should be developed to address MEs and improve patient safety in hospital settings in Iran.

## INTRODUCTION

Medical errors are an inevitable part of the healthcare system and pose a substantial threat to patient safety [1,2]. Medication errors (MEs) are one of the most common types of medical errors in healthcare systems (10 to 18% of total medical errors) and can cause serious harm to patients, even leading to death [3,4]. MEs are defined as any preventable events that may lead to the improper use of medications or may harm patients while medications are in the control of healthcare workers, patients, or consumers [5,6]. These errors can happen at any step of the medication process (prescription, transcription, dispensing, and administration) where physicians, pharmacists, and nurses are involved. Based on the findings of previous studies [7-9], MEs most commonly occur during the administration step of the medication process (i.e., giving medicine to patients), accounting for 87% of all MEs. Most MEs occurring at this step are committed by nurses. According to previous studies [3,9-13], the most prevalent administration errors are the wrong injection time, administering a medication to the wrong patient, using the wrong dose in the injection, administering the wrong drug, and administering the drug through the wrong route (e.g., intravenous injections instead of intramuscular injections). Other common medication administration errors include failing to order a drug, lack of a drug form, and failing to administer the drug [14].

The financial and non-financial burdens of MEs on the health system are significant. For example, the annual number of deaths occurring due to MEs in the US has been estimated as 44,000 to 98,000, which is greater than the number of deaths due to breast cancer, road traffic accidents, and acquired immune deficiency syndrome [5,15]. MEs lead to longer hospital stays and greater healthcare spending. The cost of hospitalization due to preventable MEs is estimated to be USD 2 billion per year in the US [11,16]. It was estimated that the mean hospitalization costs of patients who experienced MEs were higher than those of patients who did not (USD 109,199 vs. USD 56,913) [17].

Nurses play a crucial role in the provision of inpatient care, and the quality of inpatient care is closely associated with their practice. In order to reduce MEs and to improve patient safety, nurses should follow the 5 principles (“rights”) of medication administration: right patient, right medication, right dose, right route, and right time [18,19]. Understanding the prevalence and determinants of MEs among nurses is an essential first step toward the safe and appropriate administration of medication. Understanding the root causes of MEs can potentially provide useful and necessary information for health policy-makers to design and implement effective interventions to reduce the negative impact of MEs on patients and the healthcare system. Although some studies [10,13] have demonstrated a high prevalence of MEs in Iran, the prevalence and determinants of MEs among Iranian nurses are poorly understood. Thus, in this study, we aimed to examine the prevalence and types of MEs, as well as barriers to reporting MEs, among nurses working in teaching hospitals affiliated with Kermanshah University of Medical Sciences (KUMS), in the west of Iran.

## MATERIALS AND METHODS

This was a cross-sectional study carried out among 500 nurses in 7 teaching hospitals affiliated with KUMS, Kermanshah, Iran. Data collection occurred over an 8-week period from September 1 to November 30, 2016. A convenience sampling method was used to select nurses who had at least 1 year of practical work experience at the hospitals. A self-constructed questionnaire was employed to collect information on the socio-demographic characteristics of nurses and their perceptions about the main causes of MEs and barriers to reporting. The validity and reliability of the study questionnaire were investigated and confirmed in other studies [3,18,20]. Content validity was confirmed through interviews with 10 experts, and reliability was confirmed through the completion of 20 questionnaires by nurses at a 2-week interval (Cronbach alpha, 0.81). The questionnaire was composed of four major sections. The first part contained questions related to the sociodemographic characteristics of the study participants, such as age, sex, marital status, educational attainment, years of work experience in nursing, shift type, type of employment, work in 1 or more hospitals, having a second unrelated job, level of satisfaction with the nursing job, and economic status. The second part consisted of 9 yes-and-no questions designed to collect information on 9 different types of MEs made by nurses within the last 3 months. In this section, there was also a question about the proportion (%) of MEs reported to the nurse manager after their occurrence. The third part included 31 questions designed to obtain nurses’ opinions on the main factors affecting MEs. These questions were grouped into 5 categories: the managerial process (7 questions); the nursing job (7 questions); social, physiological, and psychological conditions of nurses (6 questions); factors relating to medicine and physicians (6 questions); and patient and ward conditions (6 questions). The last part of the questionnaire contained 11 questions designed to assess nurses’ views on the main barriers to reporting MEs. The third and fourth parts of the questionnaire were scored using a 5-point Likert scale, with scores ranging from 1 being ‘not at all important’ to 5 being ‘extremely important.’ The mean score for each scale was calculated by dividing the sum of scores for all questions in each scale by the number of the relevant questions. In each scale, higher scores represented a greater degree of influence on MEs. Negative binomial regression was used to identify the main predictors of the frequency of MEs among nurses. We used the incidence rate ratio with 95% confidence intervals (CIs) to assess the associations between dependent and explanatory variables. The p-values < 0.05 were defined as indicating statistical significance. All analyses were performed in Stata version 12 (StataCorp, College Station, TX, USA).

### Ethics statement

The study protocol was reviewed and approved by the Ethics Committee of the Deputy of Research, KUMS (KUMS.REC. 3004602). Verbal consent, as approved by the Deputy of Research of KUMS, was obtained from each participant after explaining the details of the study, including its purpose. It was explained that participants had the right to withdraw from the study or interrupt at any point during the data collection process.

## RESULTS

The sample consisted of 500 nurses, of whom 328 (65.6%) were female and 172 (34.4%) were male. The average age of the nurses in our sample was 31.9 years, with a standard deviation (SD) of 6.5 years. Their mean nursing experience was 5.05 years (SD, 1.02 years). Most of the nurses in our sample had a bachelor’s degree (n= 453, 90.6%). Approximately 19.0% (n= 94) of the study population were working in more than one hospital and 10.0% (n= 50) of nurses had an unrelated second job. Approximately 51.0% (n= 255) of the nurses were satisfied with their career as a nurse. Most of the nurses (n= 420, 84.0%) reported working in a rotating shift system. The descriptive statistics of the socio-demographic characteristics of the study population are presented in Table 1.

**Figure 1.**
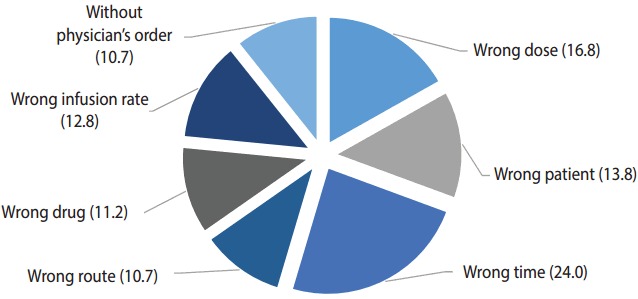
Proportion of different types of medication errors (MEs) as a percentage of total MEs among nurses in Kermanshah, west of Iran, 2016 (n = 500): nurse’s own assessment.

Based on the nurses’ own assessment, the prevalence of ME occurrence among nurses within the past 3 months was 17.0% (95% CI, 13.7 to 20.3%). Figure 1 shows the percentages of different types of MEs among the total number of MEs reported by nurses over the study period. As illustrated in Figure 1, the most common type of ME was administering a medication at the wrong time (24.0%), followed by administering the wrong dose (16.8%) and administering a medication to the wrong patient (13.8%).

Table 2 reports nurses’ views on the main causes of MEs. As shown in the Table 2, fatigue caused by excessive work hours (mean score [based on a 5-point Likert scale from 1= not at all important to 5= extremely important], 3.94± 1.02) and type of shift work (mean score, 3.74±1.05) were the two most important factors causing MEs among nurses.

Our study showed that 55% of nurses reported their MEs. Table 3 presents nurses’ opinions about the main barriers to reporting MEs. Based on our results, the most important barrier to reporting MEs was a heavy workload due to a high number of patients (3.57±1.03), followed by concerns about the consequence of MEs for patients (3.56±1.04) and concerns about the reaction of the nurse manager to MEs (3.42±1.08).

Table 4 presents the results of the negative binomial regression analysis. The results indicate the effect of explanatory variables on the number of MEs made by nurses. The regression analysis demonstrated that being male, having an unrelated second job, and fixed shift work were significantly associated with an increased number of MEs made by nurses.

## DISCUSSION

Medical errors are an inevitable part of the healthcare system. MEs are one of the most common types of errors among healthcare professionals (especially nurses), and pose a significant threat to patient safety and the quality of healthcare. In this study, we investigated various issues related to MEs among nurses working in all teaching hospitals affiliated with KUMS in 2016. Based on the nurses’ own assessments, the prevalence of MEs was found to be 17.0%. This estimated prevalence of MEs among nurses is similar to the prevalence of 16.7% observed in hospitals in Sanandaj, Iran [21]. Using observational methods in the US, Tisdale [22] also found that the prevalence of MEs in an intensive-care nursery was 17.4%. In contrast to our finding, Sarhadi et al. [4] showed that approximately 28% of nurses working in teaching hospitals affiliated with Zanjan University of Medical Sciences in Iran made at least 1 ME in 2015. The estimated prevalence of MEs was 64.5% among nurses working in a teaching hospital affiliated with Tehran University of Medical Sciences in Tehran (the capital of Iran) [23]. Higher prevalence of MEs have been reported in some international studies. For example, a study by Mrayyan et al. [6] investigated the perceptions of nurses about various issues related to MEs in Jordan and showed that the prevalence of MEs was 42.1%. The lower estimated prevalence of MEs among nurses in our study than has been observed in other similar studies in Iran can be explained by the fact that we measured the prevalence over a 3-month period, while other studies calculated the prevalence over 6 months.

Similar to previous studies in Iran [3,24-26], our study demonstrated that the most common types of MEs among patients were administering medication at the wrong time, in the wrong dose, and to the wrong patient. A study by Tang et al. [9] likewise found that administering the wrong dose (36.1%), using the wrong drug (26.4%), and administering the drug at the wrong time (18.1%) were the 3 most common types of MEs in Taiwan. Another study in Denmark indicated that the most common types of MEs among nurses were use of medication without a physician’s order, using the wrong drug, and administering the wrong dose [14].

A heavy workload was identified as the most important barrier to reporting MEs among nurses in our sample. This finding is consistent with those of previous studies [15,23,27]. For example, studies in Iran [25,28], Taiwan [9], and Germany [29] have suggested a heavy workload to be the main cause of MEs. Based on previous studies [9,19,30,31] that examined MEs from the nurses’ perspective [9,19,30,31], insufficient training, overcrowded and noisy environments, phone call orders by physicians, poor or damaged labels/packaging of medication, difficult and illegible physicians’ orders, insufficient nurse-to-patient ratios, physical and mental problems among nurses, and unfamiliarity with medications are other factors contributing to MEs. The low nurse-to-population ratio was highlighted in previous studies conducted in hospitals in Kermanshah province and other Iranian provinces. For example, Rezaei et al. [32] showed that the total number of nurses per 10,000 population in Kermanshah was 5.8, while this figure was 12.6 in Iran as a whole [33] and 6.7 in Tehran province, the capital province of Iran [34].

There is an ME reporting system in the wards of all hospitals in Iran, and when an ME occurs, nurses are asked to report the patient’s name, the type of the ME, the reason for the ME, and the potential side effects of the ME. Although nurses are asked to complete anonymous ME reports, we found that 45% of nurses did not report their MEs to their nurse managers. Based on the nurses’ opinions, the most important barriers to reporting MEs were a heavy workload due to the high number of patients, concerns about the consequence of MEs (e.g., drug side effects), and concerns about the reaction of the nurse manager. A study by Mirzaei et al. [18] showed that only 28.9% of nurses working in teaching hospitals in Kermanshah (22 out of a total sample of 95) reported their MEs to their nurse manager. Other studies in Jordan [6] and the US [35] likewise showed that the prevalence of reporting MEs to nurse managers was 42.1 and 45.6%, respectively. It is evident that the low reporting rate of MEs by nurses is a serious concern that warrants special attention from health policy-makers and hospital management.

The common causes of not reporting or underreporting MEs vary across different healthcare delivery settings. Chiang & Pepper [36] found that fear, difficulty of the reporting process, and administrative obstacles were the major causes of underreporting MEs. Concerns about the reactions of nurse managers and coworkers, fear of being blamed, and fear of the negative consequence of MEs on patients were stated as other reasons for not reporting MEs [37-39]. An unclear definition of errors among nurses is another major cause of non-reporting or underreporting MEs. The existing literature indicates that 16% of nurses do not know the accurate definition of an ME and that 14% do not know when an ME should be reported [37,38]. Some studies [38,40] have suggested that 95% of MEs were not reported by nurses due to fear of punishment. One of the reported causes of non-reporting or underreporting MEs was related to the nurse managers’ perceptions of the effect of reporting MEs. If nurse managers are concerned about the reputation of their hospitals, they may not be willing to report MEs [40,41]. However, since reporting MEs is considered an ethical responsibility and leads to improvements in the quality of healthcare and increased patient safety, healthcare professionals (especially nurse managers and nurses) should have a positive attitude toward reporting MEs. In this regard, an important step is to increase the awareness of healthcare professionals about MEs and the importance of reporting them. Furthermore, attempts should be made to address some other barriers to the improvement of patient safety through a reduction of MEs. These include barriers such as a blame culture and lack of peer-review protection [42]. To foster a patient safety culture in Iranian hospitals, reductions in the rate of non-reporting could be used as an indicator of patient safety culture.

Our empirical analysis indicated that being male, having a second unrelated job, and the type of shift work were the 3 predictors of the frequency of MEs. Previous studies [7,19,22] have also shown statistically significant associations between MEs and shift work. Shift-related fatigue among nurses working during the night and early morning can be a contributing factor to high ME rates among nurses on fixed shifts. The higher frequency of MEs among nurses with a second job may be explained by the fact that working a second job for supplemental income leads to exhaustion and fatigue among nurses, which, in turn, leads to a higher frequency of MEs among this group of nurses. Yousefi et al. [13] and Hajibabaee et al. [43] suggested a positive association between being male and MEs. Our study did not show any significant association between the frequency of MEs and nurses’ age, marital status, socioeconomic status, level of educational attainment, or years of experience. Recent studies [2,6,35,38] have likewise not suggested any associations between age or years of experience and MEs.

Our study is subject to several limitations. First, this study was carried out among nurses working in teaching hospitals in the city of Kermanshah, in the west of Iran; thus, the results of our study may be not generalizable to other types of hospitals (private hospitals and social security hospitals) or the rest of Iran. Secondly, we used self-reported data to understand the overall picture of MEs, and this method has poorer accuracy than follow-up and observational studies.

In conclusion, due to the negative consequences of MEs on the quality of care, patient safety, and performance of hospitals, nursing managers in Iran should intervene to prevent and reduce MEs among nurses. As a heavy workload was the main factor affecting MEs and the non-reporting or underreporting of MEs in our study, reducing the working hours of nursing staff can play a major role in reducing MEs among staff and in facilitating proper reporting. In addition, nursing and hospital managers should hold periodic retraining courses on the proper and safe use of a variety of medications for nurses to reduce the frequency of MEs among nurses. Moreover, since our results indicated that being male, having an unrelated second job, and fixed shift work were significantly associated with increased ME rates among nurses, nursing and hospital managers should pay more attention to the role of these factors in MEs among nurses. Considering that approximately 32 to 69% of MEs are partially or completely avoidable [11], appropriate strategies (e.g., reducing nursing staff workload) should be developed to reduce MEs and to reduce barriers to reporting MEs among nurses working in hospital settings in Iran.

## Figures and Tables

**Table 1.** Demographic, practice-related, and socioeconomic characteristics of nurses in Kermanshah, western Iran (n = 500)

Variables	n	%
Socio-demographic characteristics		
Age (yr)		
<25	85	17.0
25-35	289	57.8
≥36	126	25.2
Mean士SD	31.9 ± 6.5		
Sex		
Male	172	34.4
Female	328	65.6
Marital status		
Single	197	39.4
Married	298	59.6
Widowed or divorced	7	1.0
Practice-related characteristics		
Years of experience		
1-4	186	37.2
≥ 5	314	62.8
Mean士SD	5.0 ± 1.0		
Worked in more than 1 hospital		
Yes	95	19.0
No	405	81.0
Satisfied with nursing career		
Yes	255	51.0
No	245	49.0
Had an unrelated second job		
Yes	50	10.0
No	450	90.0
Shift type		
Rotating	420	84.0
Fixed	80	16.0
Socioeconomic characteristics		
Employment status		
Full-time	385	77.0
Part-time	65	23.0
Educational attainment		
Bachelor's degree	453	90.6
Master's degree	47	9.4
Socioeconomic status		
Poor	51	10.2
Middle	363	72.6
High	86	17.2

**Table 2.** Nurses’ assessments of the main causes of MEs among nurses in Kermanshah, western Iran (n = 500)

Categories	Items	Mean	SD
Managerial process	Lack of monitoring and supervisory mechanism for the healthcare process	2.58	1.07
	Shortages of nursing staff on the wards	3.51	1.3
	Lack of recording and reporting mechanism for MEs	2.88	1.14
	Inappropriate communication between nurses and their mangers	2.82	1.18
	Unmotivated nurses because of discrimination in the workplace	3.27	1.24
	Change in Kardex when patients are transferred to other wards	2.97	1.17
	Lack of drug information in wards	2.83	1.09
	Total	2.98	0.81
Nursing job	Failure to properly convey physicians' order to Kardex	2.70	1.23
	Give a medication at the wrong time	2.63	1.11
	Give wrong dose of a medication	2.52	1.18
	Give inaccurate diffusion rate of medication	2.56	1.15
	Use the wrong route of mediation	2.54	1.18
	Wrong patient receiving the drug	2.45	1.28
	Lack of adequate knowledge about medication	2.58	1.16
	Total	2.57	0.95
Social, physiological, and psychological conditions of nurses	Job dissatisfaction	3.29	1.19
	Economic problems	3.57	1.05
	Family problems	3.52	1.03
	Psychological and emotional problems	3.65	1.06
	Fatigue caused by excessive work hours	3.94	1.02
	Type of shift work	3.74	1.05
	Total	3.62	0.81
Medicine and physicians	Inappropriate labeling of medications	3.09	1.16
	Look-alike medications	3.12	1.08
	Availability of a variety of mediations in wards	3.07	1.07
	Phone call order by physicians	3.13	1.10
	Physician's written orders (prescriptions) is difficult to read	3.49	1.12
	Total	3.18	0.87
Patient and ward conditions	Inappropriate behaviors of patients	3.57	1.07
	Presence of patients' companions in wards	3.66	1.04
	Higher number of patients with severe illnesses in wards	3.71	0.98
	Environmental conditions of wards (e.g., light, ventilation and temperature)	3.24	1.12
	Excessive high noise levels in wards	3.34	1.07
	Drug arrangement on the shelves	3.09	1.12
	Total	3.43	0.76

ME, medication error; SD, standard deviation.

**Table 3.** Nurses’ assessments of the main barriers to reporting MEs in Kermanshah, western Iran, 2016 (n = 500)

Type of barrier	Mean	SD
Lack of information about how to report MEs	2.27	1.01
Forget to report MEs to the nurse manager	2.59	1.08
Attitude and personality of nurses	3.13	1.07
Heavy workload due to the high number of patients	3.57	1.03
Fear of disciplinary punishment	3.39	1.05
Afraid of the reaction from coworkers	3.38	1.05
Not giving priority to report after occurring MEs	3.12	1.06
Concern about the reaction of the nurse manager	3.42	1.08
Worry about the consequence of MEs (e.g., drug side effects)	3.56	1.04
Concerns about the effect of MEs on individual's earnings	3.37	1.11
Lack of clarity about the definition of MEs	2.77	0.98

ME, medication error; SD, standard deviation.

**Table 4.** Results of negative binomial regression analysis of the frequency of medication errors among nurses in Kermanshah, western Iran (n = 500)

Explanatory variables	Unadjusted	Adjusted
Socio-demographic characteristics		
Age (yr)	1.01 (0.99, 1.03)	1.02 (0.99, 1.05)
Sex		
Female	1.00	1.00
Male^*^	2.30 (1.76, 3.03)	2.16 (1.63, 2.87)
Marital status		
Single	1.00	1.00
Married	1.01 (0.57, 1.81)	1.13 (0.58, 2.20)
Practice characteristics		
Years of experience		
1-4	1.00	1.00
≥5	1.01 (0.76, 1.33)	0.83 (0.56, 1.21)
Worked in more than 1 hospital		
Yes	1.00	1.00
No	1.04 (0.74, 1.49)	1.20 (0.82, 1.74)
Satisfied with nursing career		
Yes	1.00	1.00
No	1.14 (0.87,1.50)	1.20 (0.92, 1.59)
Had an unrelated second job		
Yes	1.00	1.00
No^*^	1.82 (1.26, 2.61)	1.72 (1.16, 2.54)
Shift type		
Rotating	1.00	1.00
Fixed^*^	2.01 (1.23, 3.26)	2.18 (1.33, 3.54)
Socioeconomic characteristics		
Educational attainment		
Bachelor's degree	1.00	1.00
Master's degree	0.64 (0.36,1.12)	0.64 (0.36, 1.13)
Socioeconomic status		
Poor	1.00	1.00
Middle	0.62 (0.24,1.55)	0.60 (0.23, 1.59)
High	0.89 (0.30, 2.62)	1.10 (0.34, 3.49)
Log likelihood	−502.23		
Pseudo-R^2^	0.06		

Values are presented as incidence rate ratio (95% confidence interval).

* p<0.05.
